# Assessing the maturity of the healthcare system for integrated care: testing measurement properties of the SCIROCCO tool

**DOI:** 10.1186/s12874-019-0704-1

**Published:** 2019-03-18

**Authors:** Liset Grooten, Hubertus Johannes Maria Vrijhoef, Stefano Calciolari, Laura Guadalupe González Ortiz, Marcela Janečková, Mirella M. N. Minkman, Dirk Devroey

**Affiliations:** 1 Department of Family Medicine and Chronic Care, Faculty of Medicine and Pharmacy, Vrije Universiteit Brussel, P.O. 103, B-1090 Brussels, Belgium; 2 Department Patient & Care, Maastricht University Medical Center, Maastricht, the Netherlands; 3 Panaxea B.V., Amsterdam, the Netherlands; 4 Institute of Economics, Università della Svizzera Italiana, Lugano, Switzerland; 5 Department of Economics and Management, University of Milan Bicocca, Milan, Italy; 6 Faculty of Social Sciences, Charles University , Prague, Czech Republic; 7 Tilburg University, TIAS School for Business and Society, Tilburg, the Netherlands; 8 Vilans, National Center of Excellence, Research & Innovation, Utrecht, the Netherlands

**Keywords:** Delivery of health care, Integrated care, Convergent validity, Internal consistency, Maturity, Scaling-up, Structural validity

## Abstract

**Background:**

The Scaling Integrated Care in Context (SCIROCCO) tool has been developed to facilitate knowledge transfer and learning about the implementation and scaling-up of integrated care in European regions. To adequately test the functionality of the tool in assessing the maturity for integrated care within regions, this study evaluated its structural validity, internal consistency and convergent validity.

**Methods:**

Exploratory factor analysis was used to investigate the structural validity of the 12-items of the SCIROCCO tool. Hereafter, the internal consistency was assessed by calculating Cronbach’s and ordinal alpha. The convergent validity was explored by testing 23 pre-hypothesized relationships between items of the SCIROCCO tool and items of an instrument measuring a similar construct.

**Results:**

Factor analysis revealed a one-factor structure. Cronbach’s alpha of the overall instrument was 0.92, ordinal alpha was 0.94. Only 30.34% of the hypotheses for testing the convergent validity were met.

**Conclusion:**

The one-factor structure is considered relevant in representing the structural validity of the SCIROCCO tool. The scale of the SCIROCCO tool shows good internal consistency. The tool (DMIC Quickscan) used to assess the convergent validity might measure a different aspect of integrated care than the SCIROCCO tool. Further research is needed to continue investigating the validity and reliability of the tool.

**Electronic supplementary material:**

The online version of this article (10.1186/s12874-019-0704-1) contains supplementary material, which is available to authorized users.

## Background

To reach more European citizens who could benefit from sustainable and efficient health and social care systems, scaling-up of good practices in integrated care is desirable. Scaling-up is, however, a difficult task as there is currently insufficient knowledge available on how to use good practices in integrated care effectively and make this knowledge applicable for adopters. The use of practical tools can support the effective implementation, scalability and transferability of integrated care initiatives in Europe.

To achieve a more structured approach for scaling-up integrated care throughout Europe, the B3 Action Group on Integrated Care of the European Innovation Partnership on Active and Healthy Ageing developed the conceptual B3 Maturity Model (B3-MM) [1]. The model consists of 12 dimensions which represent the range of activities that need to be managed in order to deliver integrated care. To further develop the B3-MM, The European Union (EU) Health Programme funded project SCIROCCO, aims to develop, test and validate the B3-MM to become a key tool in facilitating exchange of good practices and scaling-up of integrated care in Europe. The definition of scaling-up which is used in the context of the EIP on AHA, and the SCIROCCO project, is derived from the review of Mangham and Hanson, and is described as “the ambition or process of expanding the coverage of health interventions,” but can also refer to “increasing the financial, human and capital resources required to expand coverage [2].” The various steps involved in the SCIROCCO project including the details on the further development of B3-MM are described in a study protocol elsewhere [3].

The SCIROCCO tool was derived from the B3-MM. The 12 dimensions, as described in the B3-MM, are displayed in the SCIROCCO tool (a full version of the tool is presented in Additional file 1). Each of the dimensions include a specific rating scale of an ordinal nature (6-points rating scale). To make the tool accessible for regions participating in the SCIROCCO project, the tool is available in English, Czech, Italian and Spanish. A healthcare region can assess its maturity for integrated care delivery, by considering each of the 12 dimensions and allocating a measure of progress or ‘maturity’ (on a 0–5 scale) to that dimension. After each of the 12 dimensions have been assessed, a simple radar diagram of the maturity status of that region is derived. This diagram provides a simple overview of a region’s strengths, weaknesses and potential improvements in integrated care. Several regions have conducted the maturity assessment. The aim of the SCIROCCO project is to match regions which indicate complementary strengths and weaknesses, to enable the exchange of knowledge and experiences between those regions. The SCIROCCO project will therefore test the process of how to ensure an appropriate flow of information and knowledge between adopting and transferring regions. This is hypothesized to be a precondition to successful transfer and scaling-up of good-practices. However, testing and validation of the SCIROCCO tool is needed first to adequately support such activities.

One part of the SCIROCCO project focuses on testing the validity and reliability of the SCIROCCO tool in separate steps. Assessing the measurement properties of the instrument, such as reliability and validity, is significant in determining the quality of the tool [4]. In a first validation study, the content-validity of the B3-MM was assessed by undertaking a literature review and Delphi study [5]. In the literature review, the 12 dimensions and assessment scales of the tool were compared with corresponding measures or instruments which were found in the literature. The outcomes showed that all the dimensions in the original version of B3-MM are aligned to the items of the corresponding instruments found in the selected literature. Thereafter, the Delphi study was undertaken. The three Delphi study rounds resulted in various amendments to the phrasing of indicators and the assessment scales used in B3-MM. In conclusion, the study showed satisfactory content-validity of the SCIROCCO tool. After the content-validity study, the SCIROCCO tool was made available as an online tool. The aim of this study is to examine the structural validity, internal consistency and convergent validity of the SCIROCCO tool. Hence the research question of this study: What is the structural validity, internal consistency and convergent validity of the SCIROCCO tool?

## Methods

The measurement properties, sample and data collection methods used for this study are presented below. Thereafter, the instruments used to assess convergent validity are described and the data analysis techniques are presented.

### Assessment of measurement properties

The measurement properties, structural validity, internal consistency and convergent validity of the SCIROCCO tool were tested in this study. Structural and convergent validity are aspects of construct validity. Construct validity ‘is based on the assumption that the measurement instrument validly measures the construct to be measured and should be assessed in case a gold standard is lacking’ [4]. The first measurement property, structural validity, is defined as ‘the degree to which the scores of a measurement instrument are an adequate reflection of the dimensionality of the construct to be measured’ [6]. This type of validity can be explored by examining the instrument’s factor structure using factor analysis. The second property, convergent validity, refers to the extent to which two instruments capture a corresponding construct [7] and can be assessed by investigating associations between these instruments. Finally, the measurement property, internal consistency, is assessed and is an aspect of reliability. It is a measure of the homogeneity of a scale and indicates the extent to which items in a scale are intercorrelated.

### Sample and data collection

#### Structural validity and internal consistency

To assess the structural validity and internal consistency of the SCIROCCO tool, subjects were invited to fill in the online SCIROCCO tool in three rounds between June 2017 and February 2018. The subjects were recruited according to the following criteria: individuals from European regions involved in the design and deployment of integrated care, including no more than 10 people per region, from several disciplines (i.e. a decision-maker, healthcare professional, an information technology specialist, regulators, payers, users group, and innovation agencies), different sectors (i.e. health care, social care, housing and voluntary sector) and different positions (i.e. senior management, front-line, back-office). In the first round, subjects were recruited from the five regions that participated in the SCIROCCO project and were recruited by SCIROCCO project members. The subjects came from the five participating European regions (Basque Country; Spain, Norrbotten; Sweden, Puglia; Italy, Olomouc; Czech Republic and Scotland). In the second round, subjects that are involved in other relevant EU projects were recruited to fill in the SCIROCCO tool. These subjects were recruited by the project coordinator and by SCIROCCO project members, mainly during dissemination activities that took place within the SCIROCCO project. In the last round, subjects were recruited by the researchers from the Vrije Universiteit Brussel. These subjects were recruited from other European regions (i.e. Denmark, France, Germany, the Netherlands, and United Kingdom) and were derived from a convenience sample (contacts provided by one of the researchers). All those who were identified and selected received a general invitation e-mail that described the purpose and procedure of the study. The invitational e-mail also included a paper providing an overview of the SCIROCCO tool and a web-link to illustrative videos and demonstrations on how to use the online version of the tool.

#### Convergent validity

To examine the convergent validity of the SCIROCCO tool, the participants who were invited in the first round were also invited to fill in the DMIC Quickscan. This occurred in a period of 6–24 weeks after the participants filled in the SCIROCCO tool. The 22 statements of the Quickscan were presented in an online survey that took about 10 min to complete. Subjects received an invitation by e-mail, including information on the survey, ethical considerations, and the link to the online DMIC Quickscan questionnaire. To construct a general profile of the subjects, data were collected about their professional position, and the name of their organisation, region and service or network.

### DMIC Quickscan

The DMIC Quickscan is based on the Development Model of Integrated Care (DMIC) questionnaire, which consists of 89 item [8]. In a recent literature review comparing the B3-MM with existing instruments that focus on assessing the development of integrated care, the DMIC was found to match with all the dimensions of the B3-MM [5]. The elements of the DMIC represent a wide range of activities considered as relevant to the realisation of integrated care which are grouped in nine clusters; ‘patient-centeredness’, ‘delivery system’, ‘performance management’, ‘quality of care’, ‘result-focused learning’, ‘interprofessional teamwork’, ‘roles and tasks’, ‘commitment’ and ‘transparent entrepreneurship’. Implementing the elements of all nine clusters contributes to the further development of integrated care. The DMIC is being used to serve as an assessment tool for health care professionals, managers and integrated care coordinators to support the implementation of improvement activities. The systematic development of the DMIC consisted of a literature study, a Delphi study and several survey studies [9]. The level of evidence on the overall quality of the measurement property content validity for the DMIC was found to be strong [5]. Moreover, the DMIC has been empirically validated in stroke, acute myocardial infarct, and dementia services in the Netherlands [10]. Furthermore, the model has been used, mainly in Europe and Canada, to evaluate and describe a variety of integration contexts [11–13].

In this study, to ensure a high response rate, we chose to use the DMIC Quickscan rather than the DMIC, due to a shorter completion time. It takes respondents10 minutes to complete the Quickscan as compared to 45 min for the DMIC. The Quickscan is extracted from the 89 items of the DMIC, of which a total of 22 items were selected based on priority scores [8] .These 22 items are presented as statements in the Quickscan, which reflect the different activities that can be undertaken to implement and develop integrated care. Subjects are asked to rate whether the description on the separate statements matches the current situation of their integrated services/network by using a 5-point scale (which ranges from fully agree-fully disagree). The DMIC Quickscan was translated into Czech, English,, Italian and Spanish by experts in the field of integrated care. Notwithstanding the theoretical validity of the DMIC and the derivation of the DMIC Quickscan from the DMIC, measurement properties including construct validity, internal consistency and convergent validity have not been tested for the DMIC nor the DMIC Quickscan. Since to our knowledge, no other similar instruments to SCIROCCO tool exist, the Quickscan was the most appropriate comparator available to test the construct validity of the SCIROCCO tool.

The convergent validity of the SCIROCCO tool was evaluated by comparing elements of the tool that used an instrument measuring a similar construct, the DMIC Quickscan. This means that the convergent validity of the SCIROCCO tool is based on comparisons between related, but not quite equivalent, concepts. The SCIROCCO tool concentrates on the maturity of elements for integrated care operating in the health care system whereas the DMIC Quickscan focuses on the development of practical elements in integrated care practices or networks. Even though both instruments are considered to operate on different levels, we expected to find a correspondence between the elements of both tools since those elements indicated to be present in the practice/network might also provide an indication of progress on these elements in the healthcare systems of those regions.

### Data analysis

Quantitative data-analysis was performed to assess the structural validity, internal consistency and convergent validity of the SCIROCCO tool. Analyses were performed using IBM SPSS Statistics software (SPSS), version 25.0.

#### Structural validity

A specialist additional module for factor analysis, R V2.4.3 was added to SPSS for the analysis of the structural validity [14]. Conventional methods of exploratory factor analysis (EFA) rely on Pearson correlations and/or maximum likelihood techniques. However, the assumptions for using these methods (item distributions that approach an equal intervals scale and a multivariate normal distribution) were not met in this study. Therefore, the polychoric correlation matrix was analysed to obtain a more accurate reproduction of the correlation structure [15]. Furthermore, EFA using minimum residual method (MINRES) of the polychoric correlation matrix was conducted to explore the structure of the items of the SCIROCCO tool. MINRES is a robust factor extraction method, as it does not require any distributional assumptions, and it can be used with small samples [16].

Multiple methods to determine the numbers of factors to extract for ordinal skewed data exist and the use of a combination of several methods is suggested [14]. In this study, two accurate techniques, Parallel Analysis (PA) [17] and Comparative Data (CD) [18], were chosen as methods to determine the number of factors to retain. Although the accuracy rates of both extraction methods decrease with smaller samples [18, 19], they are the most accurate methods known [20–22]. PA was applied using random column permutations of real data matrix, factor estimation, polychoric correlation matrix and mean eigenvalue criterion [19], a 1000 datasets were simulated. For CD, Spearman rank order correlation matrix was used to fit the ordinal scale [14]. The items of the tool relating to ‘maturity for integrated care’ were expected to be correlated, therefore oblique rotation was selected as the rotation technique. A factor loading of > 0.35 was applied.

Descriptive statistics were used to characterize the study sample. To check whether the dataset was suitable for factor analyses, Bartlett’s test for sphericity and the Kaiser–Meyer–Olkin (KMO) measure of sampling adequacy were assessed [23] Furthermore, the data were screened for any invalid data patterns (e.g., selection of “0”s for all questions), skewness and missing values. We decided to exclude items with an extreme skewed distribution (> 90% of all the responses in one category) for the analyses. Items with a high non-response (> 5% missing values) were also excluded.

#### Internal consistency

After the factor analysis was completed, the internal consistency of the tool was assessed using Cronbach alpha and ordinal alpha coefficients. Theoretically, the Cronbach alpha is only appropriate when variables are continuous, and it has been shown that Cronbach-α is negatively biased when it is used to measure the reliability of ordinal variables [20]. However, this measure is often used in practice and leads to valid results despite data that are highly skewed. In the event that the assumption of normality is violated, the ordinal alpha coefficient has been recommended as a more appropriate estimate of reliability than Cronbach’s alpha [24]. However, Chalmers indicates that coefficient α has never required continuous item-level data and that ordinal alpha should not be reported as a measure of a tests reliability, but instead should be understood as hypothetical tool [25]. Therefore, the internal consistency of each factor was examined by calculating both the Cronbach’s alpha and the ordinal reliability alpha.

#### Convergent validity

After the SCIROCCO tool and DMIC Quickscan were administered, quantitative data analysis was used to compare the items of the instruments. The convergent validity of the items of the SCIROCCO tool was evaluated by testing whether scores on the items of the SCIROCCO tool were positively associated with scores on the corresponding items of the DMIC Quickscan. Hypotheses were formulated where we expected moderate correlations between items of the two instruments. This expectation was based on the correspondence between descriptions of items of the SCIROCCO tool and the descriptions of items of the DMIC Quickscan. This resulted in the testing of 23 predefined hypotheses (see Additional file 2). Not all 22 items of the Quickscan were included in the formulated hypotheses, since some item descriptions did not correspond to any of the 12 items of the SCIROCCO tool. Correlations were calculated to test the hypothesized relationships. Strong correlations were not expected a priori because the two instruments do not measure identical constructs. Correlations falling within the range 0.30–0.50 were considered low, within the range 0.50–0.70 were considered moderate and within the range 0.70–0.90 were considered high [26]. Since the distribution of the data was skewed, the agreement between the items of the SCIROCCO tool and the DMIC Quickscan instrument were assessed using Spearman’s ρ correlation coefficients. To provide an indication of the significance and size of a statistical effect, it is recommended to use confidence limit estimation [27]. Therefore, bias-corrected accelerated (BCa) confidence intervals (CI, 95%) were computed using bootstrapping (1000 samples) for all intervals. This technique has been advised in situations where parametric assumptions are not met [28, 29].

## Results

### Factor analysis

A total of 69 respondents filled in the SCIROCCO tool. Of these, one questionnaire (1.3%) was excluded since it was incomplete. The respondents came from 13 different European countries. A large proportion of the respondents is active in the health sector (70.6%) and work mainly in management (33.8%) or as a health professional (23.5%). The characteristics of all the respondents are shown in Table 1.

**Table 1:** Characteristics of participants who completed the SCIROCCO tool (*n* = 68)

	*n* (%)
Residential country	
Belgium	1 (1.5)
Czech Republic	9 (13.2)
Denmark	1 (1.5)
Estonia	1 (1.5)
France	1 (1.5)
Greece	2 (2.9)
Hungary	2 (2.9)
Italy	11 (16.2)
The Netherlands	3 (4.4)
Poland	4 (5.9)
Spain	13 (19.1)
Sweden	8 (11.8)
United Kingdom	12 (17.6)
Healthcare system	
Asturias, Spain	3 (4.4)
Basque Country, Spain	10 (14.7)
Czech Republic	9 (13.2)
Greece	2 (2.9)
Lombardy, Italy	1 (1.5)
Netherlands	3 (4.4)
Norrbotten, Sweden	8 (11.8)
Puglia, Italy	10 (14.7)
Scotland	10 (14.7)
Other	11 (16.2)
Sector	
Health	48 (70.6)
Health, Social Care	11(16.2)
Social Care	2 (2.9)
Social Care, Voluntary	1 (1.5)
Voluntary	2 (29)
Others	4 (5.9)
Role	
Care Professional	6 (8.8)
Health Administrator	3 (4.4)
Health Economist	1 (1.5)
Care Administrator	1 (1.5)
Health ICT	5 (7.4)
Health Professional	16 (23.5)
Management	23 (33.8)
Regulator	1 (1.5)
Other	12 (17.6)

In Table 2, the item distributions are presented per item of the SCIROCCO tool. The distribution of the data over the items was non-normal and one item response was missing to the item, Evaluation Methods (1.4%). The proportion of responses per items in one answer category did not exceed the > 90% threshold.

**Table 2:** Item distributions per item of the SCIROCCO tool (The abbreviations of the items are fully described in Table 3)

Item distributions	RtC	S&G	ICT&eHealth	S&S	Funding	RoI	PA	CE	EM	BoA	IM	CB
Answer category												
0	1	10	14	7	6	2	7	7	13	7	3	7
1	17	5	14	20	23	39	29	13	13	12	14	17
2	17	18	22	17	14	9	12	24	12	6	28	16
3	22	19	12	15	14	15	9	20	20	16	17	21
4	8	7	5	8	8	3	8	4	8	18	5	2
5	3	9	1	1	3	0	3	0	1	9	1	5
Median	2	3	2	2	2	1	1	2	2	3	2	2
Total	68	68	68	68	68	68	68	68	67	68	68	68
% missing values	0	0	0	0	0	0	0	0	1,4	0	0	0
Kurtosis	−0,543	−0,680	−0,536	−0,727	-0,657	-0,559	-0,411	-0,541	− 1093	− 1057	0.134	−0,251
Skweness	0.250	−0,068	0,261	0,239	0,430	0,790	0,724	−0,255	− 0,036	− 0,347	0,209	0,343

The respondent with one item response missing was excluded in the item analysis, thus the final sample size used for the analysis was *n* = 67. In terms of the suitability of factor analysis for this dataset, Bartlett’s test for sphericity was significant (χ^2^ = 558.549, < .000), while the KMO statistic of .873 demonstrated a good sampling adequacy.

EFA was carried out on the matrix of polychoric correlations (two-step) to examine the dimensional structure underlying the SCIROCCO tool. The PA and CD techniques identified a one-factor structure of the instrument, explaining 55.57% of the variance. All the 12 items showed high factor loadings (> 0.60) to the identified factor (Table 3).

**Table 3:** Factor loadings (unrotated) of the SCIROCCO tool on one factor

	F1
Capacity Building (CB)	,866
Structure and Governance (S&G)	,823
Evaluation Methods (EM)	,785
Standardisation and Simplification (S&S)	,785
Removal of Inhibitors (RoI)	,771
Citizen Empowerment (CE)	,757
Funding	,726
Innovation Management (IM)	,721
Readiness to Change (RtC)	,702
Population Approach (PA)	,698
Breadth of Ambition (BoA)	,651
ICT and eHealth services (ICT and eHealth)	,626

### Reliability

The factor showed a Cronbach’s alpha of 0.92, and the ordinal alpha coefficient score was 0.94, presenting a high internal consistency level for the 12 items.

### Convergent validity

A total of 36 responses were collected using the DMIC Quickscan. Four respondents did not complete the full Quickscan and an additional four respondents were excluded as their matching replies to the SCIROCCO tool were not traceable due to an incorrect name. Therefore, a total of eight responses were excluded from the analyses. The characteristics of the 28 respondents are presented in Table 4.

**Table 4:** Characteristics of participants (*n* = 28) who completed the SCIROCCO tool and DMIC Quickscan

	*n* (%)
Residential country	
Czech Republic	5 (17.9)
Italy	10 (35.7)
Spain	6 (21.4)
Sweden	3 (10.7)
United Kingdom	4 (14.3)
Sector	
Health	18 (64.3)
Health; Social Care	4 (14.3)
Other(s)	2 (7.1)
Social Care	2 (7.1)
Voluntary	2 (7.1)
Role	
Care Professional	4 (14.3)
Health Administrator	1 (3.6)
Health ICT	2 (7.1)
Health Professional	4 (14.3)
Management	11 (39.3)
Other	6 (21.4)

Table 5 shows that 7 out of the 23 hypothesized relationships between the SCIROCCO tool and items of the DMIC tool were confirmed by showing moderate correlations. All the three positive hypothesized relationships between the Structure and Governance item of the SCIROCCO tool and three items of the Quickscan showed a moderate relationship. Furthermore, moderate correlations were found between Information & eHealth services, Citizen Empowerment, Evaluation Methods and Breadth of Ambition and their hypothesized relationships with items of the Quickscan. Low correlations (0.3–0.5) were found between the items of both tools in 13 of the16 remaining hypotheses. Only three of those low correlations were found to be significant.

**Table 5:** Hypothesized relationships between the items of the SCIROCCO tool and DMIC Quickscan

Hypothesis	SCIROCCO tool items	Median (IQR)	DMIC Quickscan statement per dimension	Median (IQR)	Spearman’s ρ and 95% BCa CI	*P*-value (2-tailed)
1	Readiness to Change	3 (1)	Commitment: 19	4 (2)	0.492 [.147–.763]	0.008*
2	Structure & Governance	2.5 (2)	Result-focused learning: 11	4 (1)	**0.594** [.237–.802]	0.001*
3	Structure & Governance	2.5 (2)	Roles and tasks: 15	4 (2)	**0.698** [.407–.865]	0.000*
4	Structure & Governance	2.5 (2)	Commitment: 20	4 (1)	**0.535** [.138–.801]	0.003*
5	Information & eHealth Services	2 (2)	Client-centeredness: 3	4 (2)	0.315 [−.137–.667]	0.103
6	Information & eHealth Services	2 (2)	Delivery system: 5	3.5 (2)	**0.502** [.196–.723]	0.007*
7	Standardisation & Simplification	2 (2)	Delivery system: 5	3.5 (2)	0.284 [−.066–.543]	0.143
8	Finance & Funding	3 (3)	Transparent entrepreneurship: 22	3 (2)	0.302 [−.119–.615]	0.119
9	Removal of Inhibitors	1 (1)	Result-focused learning: 12	3.5 (2)	0.240 [−.107–.554]	0.219
10	Removal of Inhibitors	1 (1)	Transparent entrepreneurship: 21	4 (1)	0.146 [−.154–.462]	0.460
11	Population Approach	1.5 (3)	Interprofessional teamwork: 13	4 (0)	0.367 [−.091–.727]	0.055
12	Citizen Empowerment	2 (2)	Client-centeredness: 3	4 (2)	**0.571** [.260–.773]	0.002*
13	Citizen Empowerment	2 (2)	Performance management: 8	3 (3)	0.474 [.092–.722]	0.011*
14	Citizen Empowerment	2 (2)	Quality of care: 10	3 (2)	0.325 [−.083–.627]	0.091
15	Evaluation Methods	2 (3)	Performance management: 6	4 (3)	0.400 [−.035–.735]	0.035
16	Evaluation Methods	2 (3)	Performance management: 7	4 (2)	**0.594** [.260–.814]	0.001*
17	Breadth of Ambition	3 (3)	Delivery system: 4	4 (1)	0.274 [−.141–.665]	0.158
18	Breadth of Ambition	3 (3)	Interprofessional teamwork: 14	4 (1)	0.320 [−.107–.702]	0.097
19	Breadth of Ambition	3 (3)	Roles and tasks: 15	4 (2)	0.367 [−.069–.743]	0.055
20	Breadth of Ambition	3 (3)	Roles and tasks: 16	4 (2)	0.334 [−.039–.658]	0.082
21	Innovation Management	2 (1)	Result-focused learning: 12	3.5 (2)	0.369 [−.104–.678]	0.054
22	Capacity Building	2.5 (1)	Performance management: 7	3.5 (2)	**0.642** [.356–.843]	0.000*
23	Capacity Building	2.5 (1)	Result-focused learning: 12	4 (2)	0.477 [.092–.744]	0.010*

Figures presented in bold show a moderate correlation (0.50-0.70)

* *p* < 0.025

## Discussion

In this study, measurement properties of the SCIROCCO tool were evaluated by examining structural validity, internal consistency and convergent validity. The findings regarding the internal structure and internal consistency provide initial support for the SCIROCCO tool. EFA supported a one-factor structure of the tool with high loadings of the items to the factor. The one-factor structure explained 55.57% of the variance in all the items. The revealed single factor is in line with the initial structure of the SCIROCCO tool, where the 12 items together were conceived to assess the one ‘underlying’ concept of maturity for integrated care. Moreover, the internal consistency, as measured with Cronbach alpha and ordinal alpha were high, thus suggesting that the different items of the SCIROCCO tool are related.

With regard to the convergent validity, slightly over one-third of the hypothesized relationships were found to be moderately correlated, thereby supporting the convergent validity of the SCIROCCO tool. The high number of low correlations between items of the two tools, however, suggests that the two instruments measure different aspects of integrated care and they should therefore not be used interchangeably. The SCIROCCO tool concentrates on the healthcare system while measuring the maturity for integrated care, while the DMIC Quickscan focuses on the presence of elements in integrated care in a practice (network). Since, to our knowledge, there is no gold standard instrument available with respect to measuring maturity for integrated care, the DMIC Quickscan included in this study was the most appropriate choice of comparative instrument that is available.

The SCIROCCO tool can be considered as a start of instrument development in assessing maturity for integrated care in the healthcare system context. However, we need to be careful in interpreting the findings since the sample size was modest, and this challenges the interpretation of the results. Furthermore, the period between the respondents filling in the SCIROCCO tool and the DMIC Quickscan varied from 6 to 24 weeks which could have also affected the outcomes. This variation was the result of the fact that the respondents were participating in the self-assessment process of the SCIROCCO project; due to workload, some regions decided to wait a bit longer to invite the respondents to complete the DMIC Quickscan to ensure a high response rate. It is therefore important to perform the analysis on a larger sample size. This to explore whether a greater correspondence among the items of the instruments can be found so as to support the convergent validity of the tool or to investigate whether the instruments do indeed measure different constructs. Since the SCIROCCO project will be followed-up by a new project focussing on maximising the value and impact of the tool, we recommend paying attention to this matter in the new project.

This study has three limitations. The first, and main, limitation is the modest sample size, which may have influenced the robustness of the factor analysis. In conducting the EFA, several aspects guided us in choosing the appropriate factor extraction method. When the sample size and number of factors are expected to be small, the use of an unweighted least squares (ULS) method to determine the factor structure is recommended [30–32]. In our study, we used MINRES, which is equivalent to ULS. MINRES is very robust and it does not require any distributional assumptions, therefore it can be used with small samples and when a correlation matrix is not positive definite [16]. In addition, calculation of the sample size necessary to assess structural validity is recommended by a subjects-to-variables ratio (“N to p” ratio, where “N” is the sample size and “p” the number of items included in analysis). The subjects-to-variables ratio of our sample 5.5:1, was considered sufficient as it falls within the range of acceptability [20]. In the literature, an acceptable ratio ranges from at least 5:1, while a 10:1 ratio is considered as rule of thumb for determining a priori sample size. To estimate the consistency of the results, we repeated the analyses using the alternative method of principal axis factoring. The analysis resulted in the same factor loadings (the results of this analysis are available via the corresponding author). Notwithstanding the acceptable subject-to-variable ratio, the use of MINRES as the extraction method, and the stable alternative analysis, the modest sample size of our study does not allow for strong conclusions about the factor solution. However, we consider the one factor solution relevant. We recommend to perform additional analyses, using confirmatory factor analyses on a similar but larger sample to test how well the measured variables confirm the underlying factor structure found in this study. To further investigate measurement properties of the tool, the SCIROCCO project planned to conduct a test-retest of the tool to test the reliability and stability of the instrument.

A second limitation of this study is that we did not conduct a nonresponse analysis and, therefore we have no specific information about the non-responders. Subjects were invited to fill in the SCIROCCO tool via three rounds, including project members of SCIROCCO, during several dissemination activities which were organized by the project consortium. Therefore, we were unable to track the response rate in the study. Several factors may have contributed to non-responses. One of these is the fact that the tool was distributed to respondents in several countries in Europe and was available in only four languages, which may have created an obstacle for some respondents to fill in the tool. Other factors could be the demands on some respondents of participating in the SCIROCCO project itself (multiple requests were made to them to respond), a lack of time, and either not feeling a specific urge to fill in the tool or seeing the immediate benefit from doing so.

A third limitation which needs to be considered is the implication for this study of the availability of the SCIROCCO tool, and the undertaking of the DMIC Quickscan, in these four languages. The tool was originally developed in English and the content-validity of the tool was assessed using this language version. Thereafter, the tool was translated, and the adequacy and clarity of this translation was checked by the consortium partners based in the different European regions. Since the context, languages and commonly used expressions of the different regions in Europe may have an influence on the description of various aspects and concepts related to integrated care, the translations could have resulted in slightly different wordings in the SCIROCCO tool itself. Furthermore, the DMIC Quickscan was also translated to English by its developer and to Czech, Italian and Spanish by mother-tongue speakers who are researchers in the field of integrated care. The translations could also have led to there being slightly different wordings used in the DMIC Quickscan. We expect that these slight differences may have resulted in the provision of different answers to the items of the SCIROCCO tool among the different regions. It is therefore recommended, as a next step, to explore the factor structure of the different language versions of the SCIROCCO tool.

## Conclusion

The SCIROCCO tool is a promising instrument which offers regions a tailored approach facilitating progress in integrated care. It provides insights into the strengths and weaknesses in integrated care on which regions can be matched and shared learning can be facilitated. Determination of the SCIROCCO tool’s measurement properties is important to ensure a valid and reliable assessment of the maturity level of the regional healthcare system. This is the first study to have assessed the structural validity, internal consistency and convergent validity of the SCIROCCO tool. The construct of the SCIROCCO tool presented one relevant underlying factor: it seems that the tool reflects the maturity of the health care system context in providing integrated care with adequate validity. The internal reliability of the one-factor structure was high. For the convergent validity, only 7 out of the 23 hypothesized relationships on the correlations between the SCIROCCO tool and the DMIC Quickscan were met. This outcome may possibly be due either to the modest sample size or the partly different focus of both tools. Further studies should therefore be conducted in larger samples of individuals involved in integrated care to confirm the validity and assess the reliability of the SCIROCCO tool.

## Additional files

Additional file 1: Full version of the SCIROCCO tool. (DOCX 181 kb)

Additional file 2: 23 predefined hypotheses on expected moderate correlations between items of the SCIROCCO tool and the DMIC Quickscan. (DOCX 30 kb)

## Abbreviations

BCa: Bias-corrected accelerated
CD: Comparative Data
CI: Confidence interval
DMIC: Development Model for Integrated Care
EFA: Exploratory factor analysis
ICT: Information and communications technology
KMO: Kaiser–Meyer–Olkin
MINRES: Minimum residual method
PA: Parallel Analysis
SCIROCCO: Scaling Integrated Care in Context
ULS: Unweighted least squares

## References

[1] Henderson D, Pavlickova A, Lewis L (2016). Scalability and transferability of good practices in Europe: what does it take?. Int J Integr Care.

[2] Mangham LJ, Hanson K (2010). Scaling up in international health: what are the key issues?. Health Policy Plan.

[3] Grooten L, Alexandru C-A, Alhambra-Borrás T, Anderson S, Avolio F, Valia Cotanda E (2018). Journal of integrated care a scaling-up strategy supporting the expansion of integrated care: a study protocol article information.

[4] de Vet HCW, Terwee CB, Mokkink LB, Knol DJ. Measurement in Medicine: A Practical Guide. New York: Cambridge University Press; 2011.

[5] Grooten L, Vrijhoef HJM, Borgermans L (2018). An instrument to measure maturity of integrated care: a first validation study. Int J Integr Care.

[6] Mokkink LB, Terwee CB, Knol DL, Stratford PW, Alonso J, Patrick DL (2010). The COSMIN checklist for evaluating the methodological quality of studies on measurement properties: a clarification of its content. BMC Med Res Methodol.

[7] Carlson KD, Herdman AO (2012). Understanding the impact of convergent validity on research results. Organ Res Methods.

[8] Minkman M, Ahaus K, Fabbricotti I, Nabitz U, Huijsman R (2009). A quality management model for integrated care: results of a Delphi and concept mapping study. Int J Qual Heal Care.

[9] Minkman M (2016). The Development Model for Integrated Care: a validated tool for evaluation and development. J Integr Care.

[10] Minkman MMN, Vermeulen RP, Ahaus KTB, Huijsman R (2011). The implementation of integrated care: the empirical validation of the development model for integrated care. BMC Health Serv Res.

[11] Zonneveld N, Vat LE, Vlek H, Minkman MMN (2017). The development of integrated diabetes care in the Netherlands: a multiplayer self-assessment analysis. BMC Health Serv Res.

[12] Vat LE, Middelkoop I, Buijck BI, Minkman MMN (2016). The development of integrated stroke Care in the Netherlands a benchmark study. Int J Integr Care.

[13] Longpré C, Dubois C-A (2015). Implementation of integrated services networks in Quebec and nursing practice transformation: convergence or divergence?. BMC Health Serv Res.

[14] Courtney MGR (2013). Determining the number of factors to retain in EFA : using the SPSS R-menu v2 . 0 to make more judicious estimations. Pract Assessment Res Eval.

[15] Holgado-Tello FP, Chacón-Moscoso S, Barbero-García I, Vila-Abad E (2009). Polychoric versus Pearson correlations in exploratory and confirmatory factor analysis of ordinal variables. Qual Quant.

[16] Jöreskog KG (2003). Factor analysis by MINRES. Chicago Sci Softw Retrieved June.

[17] Horn JL (1965). A rationale and test for the number of factors in factor analysis. Psychometrika..

[18] Ruscio J, Roche B (2012). Determining the number of factors to retain in an exploratory factor analysis using comparison data of known factorial structure. Psychol Assess.

[19] Garrido LE, Abad FJ, Ponsoda V (2013). A new look at horn’s parallel analysis with ordinal variables. Psychol Methods.

[20] Costello AB, Osborne JW (2005). Best practices in exploratory factor analysis : four recommendations for getting the Most from your analysis. Pract Assessment Res Educ.

[21] Gaskin CJ, Happell B (2014). On exploratory factor analysis: a review of recent evidence, an assessment of current practice, and recommendations for future use. Int J Nurs Stud.

[22] Basto M, Pereira JM (2012). An SPSS R -menu for ordinal factor analysis. J Stat Softw.

[23] Thompson B (2004). Exploratory and confirmatory factor analysis: understanding concepts and applications.

[24] Zumbo BD, Gadermann AM, Zeisser C (2007). Ordinal versions of coefficients alpha and Theta for Likert rating scales. J Mod Appl Stat Methods.

[25] Chalmers RP. On misconceptions and the limited usefulness of ordinal alpha. Educ Psychol Meas. 2017;78(6):1056–71. 10.1177/0013164417727036 PMC6293415 30559513

[26] Hinkle DE, Wiersma W. Applied Statistics for the behavioral sciences. Boston: Houghton Mifflin; 2002.

[27] MacKinnon DP, Lockwood CM, Williams J (2004). Confidence limits for the indirect effect: distribution of the product and resampling methods. Multivariate Behav Res.

[28] Chan W, Chan DW-L (2004). Bootstrap standard error and confidence intervals for the correlation corrected for range restriction: a simulation study. Psychol Methods.

[29] Efron B, Tibshirani RJ. An Introduction to the Bootstrap. New York, NY Chapman & Hall. 1993.

[30] Sapnas KG, Zeller RA (2002). Minimizing sample size when using exploratory factor analysis for measurement. J Nurs Manag.

[31] de Winter JCF, Dodou D, Wieringa PA (2009). Exploratory factor analysis with small sample sizes. Multivariate Behav Res.

[32] Jung S (2013). Exploratory factor analysis with small sample sizes: a comparison of three approaches. Behav Process.

