# Supplementary material for: Assessing the maturity of the healthcare system for integrated care: testing measurement properties of the SCIROCCO tool
Source: BMC Med Res Methodol. 2019 Mar 18;19:63. doi: 10.1186/s12874-019-0704-1 (PMC6423766; doi:10.1186/s12874-019-0704-1)
Supplement: Supplementary file 2 — 23 predefined hypotheses on expected moderate correlations between items of the SCIROCCO tool and the DMIC Quickscan. (DOCX 30 kb) [file 12874_2019_704_MOESM2_ESM.docx]

**Hypotheses’ SCIROCCO tool vs DMIC Quick scan**

We hypothesise that there are correlations between the items of the SCIROCCO tool presented in the left column and the items of the DMIC Quick scan displayed in the right column. Higher scores on the subscales about the items of the SCIROCCO tool in the right column, would be highly positively associated with scores on items of the DMIC Quickscan in the left column.

|  |  |
| --- | --- |
| ITEMS SCIROCCO tool | ITEMS DMIC QUICK SCAN PER DIMENSION |
| **1. Readiness to Change**  Objectives: If the existing systems of care need to be re-designed to provide a more integrated set of services, this will require change across many levels, the creation of new roles, processes and working practices, and new systems to support information sharing and collaboration across care teams. This will be disruptive and may be viewed negatively by workers, press and public, so a clear case needs to be made for those changes, including a justification, a strategic plan, and a vision of better care.  •Creating a compelling vision, with a real sense of urgency, and enlisting stakeholder support including political leadership, management, care professionals, public and press.  •Accepting the reality that care systems are unsustainable and need to change.  •Publishing a clear description of the issues, the choices that need to be made, and the desired future state of the care systems, stating what will be the future experience of care.  •Creating a sense of urgency to ensure sustained focus, and building a ‘guiding coalition’ for change.  Assessment scale:  0 – No acknowledgement of compelling need to change  1 – Compelling need is recognised, but no clear version or strategic plan  2 – Dialogue and consensus-building underway; plan being developed  3 – Vision or plan embedded in policy; leaders and champions emerging  4 – Leadership, vision and plan clear to the general public; pressure for change  5 – Political consensus; public support; visible stakeholder engagement. | **Commitment:**  19. Senior leaders of the involved partner organisations are in support of  the aims. |
| **2. Structure & Governance**  Objectives: The broad set of changes needed to deliver integrated care at a regional or national level presents a significant challenge. It needs multi-year programmes with excellent change management, funding and communications, and the power to influence and (sometimes) mandate new working practices. This means alignment of purpose across diverse organisations and professions, and the willingness to collaborate and put the interest of the overall care system above individual incentives. It also means managing the introduction of eHealth services to enable integrated care in a way that makes them easy to use, reliable, secure, and acceptable to care professionals and citizens alike.  •Enabling properly funded programmes, including a strong programme, project management and change management; establishing ICT or eHealth competence centres to support roll-out; distributed leadership, to reduce dependency on a single heroic leader; excellent communication of goals, progress and successes.  •Managing successful eHealth innovation within a properly funded, multi-year transformation programme.  •Establishing organisations with the mandate to select, develop and deliver eHealth services.  Assessment scale:  0 – Fragmented structure and governance in place  1 – Recognition of the need for structural and governance change  2 – Formation of task forces, alliances and other informal ways of collaborating  3 – Governance established at a regional or national level  4 – Roadmap for a change programme defined and broadly accepted  5 – Full, integrated programme established, with funding and a clear mandate. | **Result-focused learning:**  INDICATORS QUICKSCAN:  11. It is clear what health and care support the partner organisations will deliver and the benefits of the collaboration for each care partner are clear.  **Roles and tasks:**  INDICATORS QUICKSCAN:  15. Clear agreements on tasks and responsibilities of the involved partner  organisations are present.  **Commitment:**  20. The different interests of the partner organisations in the collaboration are being taken into account, and collaboration is based on trust. |
| **3. Information & eHealth Services**  Objectives: Integrated care requires, as a foundational capability, sharing of health information and care plans across diverse care teams that leads progressively to systems for enabling continuous collaboration, measuring and managing outcomes, and enabling citizens to take a more active role in their care. This means building on existing eHealth services, connecting them in new ways to support integration, and augmenting them with new capabilities, such as enhanced security and mobility.  •Essential components to enable information-sharing, based on secure and trusted services.  •‘Digital first’ policy (where possible, move phone and face-to-face services to digital services to reduce dependence on staff and promote self-service).  •Availability of fundamental building blocks to enable eHealth and eServices (‘infostructure’).  •Confidentiality and security designed into patient records, registries, online services etc.  •Enabling of new channels for healthcare delivery to replace face-to-face and telephone contact.  Assessment scale:  0 – Information systems are not designed to support integrated care  1 – Information and eHealth services to support integrated care are being piloted  2 – Information and eHealth services to support integrated care are deployed but there is not yet region wide coverage  3 – Information and eHealth services to support integrated care are available via a region-wide service but use of these services is not mandated  4 – Mandated or funded use of regional/national eHealth infrastructure across the healthcare system  5 – Universal, at-scale regional/national eHealth services used by all integrated care stakeholders. | **Client-centeredness**:  INDICATORS QUICKSCAN:  3. Partner organisations ensure that the offered information is accessible and  relevant to communication styles and needs of clients.  **Delivery system:**  INDICATORS QUICKSCAN:  5. Partner organisations have made agreements on procedures for the  exchange of client information and how to work with or share electronic client  records. |
| **4. Standardisation & Simplification**  Objectives: When considering eHealth services and how they can support the information sharing and collaboration needs of integrated care, the task can be made easier if the number of different systems in use, and the formats in which they store data, can be simplified. Practically, this means trying to consolidate data centres, standardising on fewer systems, and agreeing on what informatics standards will be used across a region or country.  •Simplification of infrastructure; fewer integration points to manage; easier interoperability.  •Consolidation of applications and data centres into fewer sites.  •Regional standardisation on fewer (or single) solutions.  •Ability to view and exchange medical data from different systems across diverse care settings.  Assessment scale:  0 – No standards in place or planned that support integrated care services  1 – Discussion of the necessity of ICT to support integrated care and of any standards associated with that ICT  2 – An ICT infrastructure to support integrated care has been agreed together with a recommended set of information standards – there may still be local variations  3 – A recommended set of agreed information standards at regional/national level; some shared procurements of new systems at regional/national level; some large-scale consolidations of ICT underway  4 – A unified set of agreed standards to be used for system implementations specified in procurement documents; many shared procurements of new systems; consolidated data centres and shared services widely deployed  5 – A unified and mandated set of agreed standards to be used for system implementations fully incorporated into procurement processes; clear strategy for regional/national procurement of new systems; consolidated datacentres and shared services (including the cloud) is normal practice. | **Delivery system:**  INDICATORS QUICKSCAN:  5. Partner organisations have made agreements on procedures for the  exchange of client information and how to work with or share electronic client  records. |
| 5. Funding  Objectives: Changing systems of care so that they can offer better integration requires initial investment and funding; a degree of operational funding during transition to the new models of care; and on-going financial support until the new services are fully operational and the older ones are de-commissioned. Ensuring that initial and on-going costs can be financed is an essential activity that uses the full range of mechanisms from regional/national budgets to ‘stimulus’ funds, European Union investment funds, public-private partnerships (PPP) and risk-sharing mechanisms).  Assessment scale:  0 – No additional funding is available to support the move towards integrated care  1 – Funding is available but mainly for the pilot projects and testing  2 – Consolidated innovation funding available through competitions/grants for individual care providers and small-scale implementation  3 – Regional/national (or European) funding or PPP for scaling-up is available  4 – Regional/national funding for on-going operations is available  5 – Secure multi-year budget, accessible to all stakeholders, to enable further service development | **Transparent entrepreneurship**:  INDICATORS QUICKSCAN:  22. Agreements on the resources and financial risks for the integrated care  service have been reached. |
| 6. Removal of Inhibitors  Objectives: Even with political support, funded programmes and good eHealth infrastructure, many factors can still make integrated care difficult to deliver, by delaying change or limiting how far change can go. These include legal issues with data governance, resistance to change from individuals or professional bodies, cultural barriers to the use of technology, perverse financial incentives, and lack of skills. These factors need to be recognised early, and a plan developed to deal with them, so as to minimise their impact.  •Actions to remove barriers: legal, organisational, financial, skills.  •Changes to the law concerning e.g., medical acts, information governance, data sharing –factors which may hold up innovation.  •Creation of new organisations or collaborations to encourage cross-boundary working (‘normative integration’).  •Changes to reimbursement to support behavioural change and process change.  •Education and training to increase understanding of ICT and speed up solution delivery.  Assessment scale:  0 – No awareness of the effects of inhibitors on integrated care  1 – Awareness of inhibitors but no systematic approach to their management is in place  2 – Strategy for removing inhibitors agreed at a high level  3 – Implementation Plan and process for removing inhibitors have started being implemented locally  4 – Solutions for removal of inhibitors developed and commonly used  5 – High completion rate of projects & programmes; inhibitors no longer an issue for service development | **Result-focused learning**:  INDICATORS QUICKSCAN:  12. A partnership culture focusing on mutual learning, knowledge exchange and improvement has been developed.  **Transparent entrepreneurship**  21. There is a commitment to a joint responsibility for the results to be achieved. |
| 7. Population Approach  Objectives: Integrated care can be developed to benefit those citizens who are not thriving under existing systems of care, in order to help them manage their health and care needs in a better way, and to avoid emergency calls and hospital admissions and reduce hospital stays. This is a practical response to meeting today’s demands. Population health goes beyond this, and uses methods to understand where future health risk (and so, demand) will come from. It offers ways to act ahead of time, to predict and anticipate, so that citizens can maintain their health for longer and be less dependent on care services as they age.  •Understanding and anticipating demand; meeting needs better.  •Improving the resilience of care systems by using existing data on public health, health risks, and service utilisation.  •Taking steps to divert citizens into more appropriate and convenient care pathways based on user preferences.  •Predicting future demand and taking steps to reduce health risks though technology-enabled public health interventions.  Assessment scale:  0 – Population health approach is not applied to the provision of integrated care services  1 – A population risk approach is applied to integrated care services but not yet systematically or to the full population  2 – Risk stratification is used systematically for certain parts of the population (e.g. high-use categories)  3 – Group risk stratification for those who are at risk of becoming frequent service users  4 –Population-wide risk stratification started but not fully acted on  5 – Whole population stratification deployed and fully implemented | **Interprofessional teamwork**:  INDICATORS QUICKSCAN:  13. The targeted client group of the care service has been defined. |
| 8. Citizen Empowerment  Objectives: Health and social care systems are under increasing pressure to respond to demands which could otherwise be handled by citizens and carers themselves. The evidence suggests that many individuals would be willing to do more to participate in their own care if easy-to-use services, such as appointment booking, self-monitoring of health status, and alternatives to medical appointments, were available to them. This means providing services and tools which enable convenience, offer choice, and encourage self-service and engagement in health management.  Assessment scale:  0 – Citizen empowerment is not considered as part of integrated care provision  1 – Citizen empowerment is recognised as important part of integrated care provision but effective policies to support citizen empowerment are still in development  2 - Citizen empowerment is recognised as important part of integrated care provision, effective policies to support citizen empowerment are in place but citizens do not have access to health information and health data  3 - Citizens are consulted on integrated care services and have access to health information and health data  4 – Incentives and tools exist to motivate and support citizens to co-create healthcare services and use these services to participate in decision-making process about their own health  5 – Citizens are fully engaged in decision-making processes about their health, and are included in decision-making on service delivery and policy-making. | **Client-centeredness:**  INDICATORS QUICKSCAN  3. Partner organisations ensure that the offered information is accessible and  relevant to communication styles and needs of clients.  **Performance management:**  INDICATORS QUICKSCAN  8. There is a systematic procedure for the evaluation of the experiences of  clients and their families.  **Quality of care:**  INDICATORS QUICKSCAN  10. People and their families are meaningfully involved in the improvement of the integrated care service (f.i. in improvement projects). |
| 9. Evaluation Methods  Objectives: As new care pathways and services are introduced to support integrated care, there is a clear need to ensure that the changes are having the desired effect on quality of care, cost of care, access and citizen experience. This supports the concept of evidence-based investment, where the impact of each change is evaluated, ideally by health economists working in universities or in special agencies. Health technology assessment (HTA) is an important method here, and can be used to justify the cost of scaling up good practices to regional or national level.  •Establishing baselines (on cost, quality, access etc.) in advance of new service introduction.  •Systematically measuring the impact of new services and pathways using appropriate methods (e.g., observational studies, incremental improvement, clinical trials).  •Generating evidence that leads to faster adoption of good practice.    Assessment scale:  0 – No evaluation of integrated care services is in place or in development  1 – Evaluation of integrated care services exists, but not as a part of a systematic approach  2 – Evaluation of integrated care services is planned to take place and be established as part of a systematic approach  3 – Some integrated care initiatives and services are evaluated as part of a systematic approach  4 – Most integrated care initiatives are subject to a systematic approach to evaluation; published results  5 – A systematic approach to evaluation, responsiveness to the evaluation outcomes, and evaluation of the desired impact on service redesign (i.e., a closed loop process). | **Performance measurement:**  INDICATORS QUICKSCAN:  6. Performance indicators and data sources have been defined to monitor  outcomes.  7. An evaluation of impact and process is carried out periodically and used to  improve implementation. |
| 10. Breadth of Ambition  Objectives: Integrated care includes many levels of integration, such as integration between primary and secondary care, of all stakeholders involved in the care process, or across many organisations. It may be developed simply for healthcare needs (i.e., vertical integration) or it may include social workers, the voluntary sector, and informal care (i.e., horizontal integration). The broader the ambition, the more numerous and diverse the stakeholders who have to be engaged. Similarly, integration may include all levels of the system or may be limited to clinical information sharing. The long-term goal should be fully integrated care services which provide a complete set of seamless interactions for the citizen, leading to better care and improved outcomes.  •Integration supported at all levels within the healthcare system – at the macro (policy, structure), meso (organisational, professional) and micro (clinical) levels.  •Integration between the healthcare system and other care services (including social, voluntary, informal, family services).  •Seamless transition for the patient between and within care services.  Assessment scale:  0 – Integrated services arise but not as a result of planning or the implementation of a strategy  1 – The citizen or their family may need to act as the integrator of service in an unpredictable way  2 – Integration within the same level of care (e.g., primary care)  3 – Integration between care levels (e.g., between primary and secondary care)  4 – Integration includes both social care service and health care service needs  5 – Fully integrated health & social care services. | **Delivery system:**  INDICATORS QUICKSCAN:  4. Partner organisations have made agreements on referral processes and  care pathways  **Interprofessional teamwork**:  INDICATORS QUICKSCAN:  14. Professionals collaborate with each other in multidisciplinary team(s).  **Roles and tasks**:  INDICATORS QUICKSCAN:  15. Clear agreements on tasks and responsibilities of the involved partner  organisations are present.  16. Professionals understand and draw on each another’s expertise and roles. |
| 11. Innovation Management  Objectives: Many of the best ideas are likely to come from clinicians, nurses and social workers who understand where improvements can be made to existing processes. These innovations need to be recognised, assessed and, where possible, scaled up to provide benefit across the system. At the same time, universities and private sector companies are increasingly willing to engage in open innovation, and innovative procurement, in order to develop new technologies, test process improvements and deliver new services that meet the needs of citizens. There is also value in looking outside the system to other regions and countries that are dealing with the same set of challenges, to learn from their experiences. Overall, this means managing the innovation process to get the best results for the systems of care, and ensuring that good ideas are encouraged and rewarded.  •Adopting proven ideas faster.  •Enabling an atmosphere of innovation from top to bottom, with collection and diffusion of best practice.  •Learning from inside the system, as well as from other regions, to expand thinking and speed up change.  •Involving universities and private sector companies in the innovation process (i.e., ‘open innovation’).  •Using innovative procurement approaches (Pre-Commercial Procurement, IPP, PPP, Shared Risk, Outcome-Based Payment)  •Using European projects (e.g., Horizon 2020, EIP, CEF).  Assessment scale:  0 – No innovation management in place  1 – Innovation is encouraged but there is no overall plan  2 – Innovations are captured and there are some mechanisms in place to encourage knowledge transfer  3 – Formalised innovation management process is planned and partially implemented  4 – Formalised innovation management process is in place and widely implemented  5 – Extensive open innovation combined with supporting procurement & the diffusion of good practice is in place | **Result-focused learning**:  INDICATORS QUICKSCAN:  12. A partnership culture focusing on mutual learning, knowledge exchange and improvement has been developed. |
| 12. Capacity Building  Objectives: As the systems of care are transformed, many new roles will need to be created and new skills developed. These will range from technological expertise and project management, to successful change management. The systems of care need to become ‘learning systems’ that are constantly striving to improve quality, cost and access. They must build their capacity so as to become more adaptable and resilient. As demands continue to change, skills, talent and experience must be retained. This means ensuring that knowledge is captured and used to improve the next set of projects, leading to greater productivity and increasing success.  •Increasing technology skills; continuous improvement.  •Building a skill base that can bridge the clinician-technologist gap and ensure that needs are understood and addressed by ICT.  •Providing tools, processes and platforms to allow organisations to assess themselves and build their own capacity to deliver successful change.  •Creating an environment where service improvements are continuously evaluated and delivered for the benefit of the entire care system.  Assessment scale:  0 – Integrated care services are not considered for capacity building  1 – Some systematic approaches to capacity building for integrated care services are in place  2 – Cooperation on capacity building for integrated care is growing across the region  3 – Systematic learning about integrated care and change management is in place but not widely  implemented.  4 – Systematic learning about integrated care and change management is widely implemented;  knowledge is shared, skills retained and there is a lower turnover of experienced staff.  5 – A 'person-centred learning healthcare system’ involving reflection and continuous improvement. | **Performance measurement:**  INDICATORS QUICKSCAN:  7. An evaluation of impact and process is carried out periodically and used to  improve implementation.  **Result-focused learning**:  INDICATORS QUICKSCAN:  12. A partnership culture focusing on mutual learning, knowledge exchange and improvement has been developed. |
